# Soil properties under different ecological restoration modes for the quarry in Yanshan mountains of Hebei province, China

**DOI:** 10.7717/peerj.14359

**Published:** 2022-11-18

**Authors:** Jianjun Ma, Chenyao Li, Liu Hui, Jing Wang, Yongjun Fan

**Affiliations:** 1 College of Life Science, Lang Fang Normal University, Lang Fang, Hebei Province, China; 2 Inner Mongolia Key Laboratory for Biomass-Energy Conversion, Baotou, Inner Mongolia, China; 3 School of Life Science and Technology, Inner Mongolia University of Science and Technology, Baotou, Inner Mongolia, China; 4 Langfang Zetong Forestry Engineering Design Co., Ltd., Lang Fang, Hebei Province, China; 5 Department of Civil Engineering, Ordos Institute Technology, Ordos, Inner Mongolia, China

**Keywords:** Quarry mining, Vegetation restoration, Soil microorganisms, Yanshan mountains

## Abstract

The ecological environment of quarry mining area is fragile, and the vegetation restoration cycle is long and difficult, so scientific and appropriate artificial vegetation is of great significance to ecological restoration. The purpose of this study was to evaluate the herbaceous and woody vegetation restoration, including *Medicago sativa* (Me), artificial miscellaneous grass (Mg), *Rhus typhina* (Rh), fruit orchard (Or) and *Pinus tabulaeformis* (Pi), to investigate the soil physicochemical properties and the structure of the microbial communities, and to reveal the correlation between them. The results addressed that *Medicago sativa* and artificial miscellaneous grass had significant effect on soil remediation, which were conducive to scientific and efficient ecological restoration, and could promote ecological restoration in the damaged ecosystems. While, the modes of Rh and Pi were not suitable for ecological restoration in this study area because they had strong allelopathy. Another arborous restoration mode of Or showed a better improvement effect (including soil nutrients, soil microbial diversity, *etc.*) than that of Rh and Pi. The findings also indicated that the herbaceous vegetation restoration modes of Me and Mg significantly increased the relative abundance of Proteobacteria, Acidobacteria, Actinobacteria bacteria, Ascomycota and Mortierllomycota fungi, and reduced the relative abundance of Firmicutes bacteria and Basidiomycota fungi. This study also revealed that the trend of bacterial localization in the fruit orchard, artificial miscellaneous grass and *Medicago sativa* was more obvious. Among many soil abiotic factors, the contents of organic matter, available nitrogen and pH were the most important factors affecting soil microbial community.

## Introduction

Quarry mining area is an extremely degraded ecosystem. Due to the stripping of the original topsoil and vegetation, the soil erosion is intensified and the ecosystem function is reduced. Vegetation restoration is the only strategy to control soil erosion and restore ecosystem function (An, Huang & Zheng, 2009), and soil quality also determines the nature of vegetation succession and the success of ecological restoration (Putten et al., 2013). Soil is the carrier of many ecological processes in the ecosystem, such as nutrient cycling, water balance and litter decomposition (Yang et al., 2014). The main obstacle to the vegetation restoration of mine wasteland is the soil factor with special and adverse properties (Wang et al., 2011). The soil structure and nutrient status play a key role in the growth of plants, directly affect the composition and physiological vitality of plant communities, determine the structure, function and productivity level of the ecosystem. And more importantly, soil is one of the key indicators to measure the restoration and maintenance of ecological functions of degraded ecosystems (Yu et al., 2007). Therefore, it is necessary to evaluate the soil quality under different vegetation restoration modes (Song et al., 2021). The monitoring of soil microorganisms provides an important basis for ecosystem restoration in mining areas to evaluate the realization of objectives (Harris, 2003; Kiehl et al., 2010; Eaton et al., 2015).

Theoretically speaking, natural restoration is the most scientific and sustainable natural integration process closest to the original ecosystem (Chazdon, 2008; Herath et al., 2009; Chiquoine, Abella & Bowker, 2016). However, in northern China, due to lack of water resources, poor soil conditions and fragile ecological environment, natural restoration usually takes a long time, and people may face long-term ecological risks (Hobbs, Higgs & Harris, 2009; Gastauer et al., 2019). Therefore, proper artificial vegetation restoration is of great significance. So far, many ecological disturbance areas, especially extreme disturbance areas such as mining, can be quickly restored by artificial restoration according to “human will”, but the restoration cost is high. The restoration of “rapid greening”, which simply pursues visual beauty, is favored by more and more people (especially decision-makers) (Ren, 2006), while the restoration of ecosystem structure and function is neglected. Therefore, the disadvantages of artificial restoration in integrating natural ecological processes and characteristics have also emerged (Lu et al., 2021).

As early as the beginning of the 20th century, developed countries began to carry out land restoration in mining areas, including classified planting, ecological landscape reconstruction of abandoned mines, environmental monitoring and supervision (Hu, 2019). In China, vegetation restoration and ecological environment management in mining areas started later. The research on vegetation restoration in mining areas focuses on the technology of vegetation restoration and reconstruction (Zhao et al., 2018), the selection of vegetation species (Wei, Zhen & Du, 2022), and the impact of vegetation restoration on other biological and non biological factors in the ecosystem (Liu, 2022), while there are few studies on the sustainability of the vegetation restoration mode and the integration with the local ecosystem (Wang et al., 2020). Under the background of sustainable development, the establishment of an efficient and sustainable artificial vegetation restoration system in mining areas has gradually attracted people’s attention (Bai et al., 2020; Yi et al., 2020). The purpose of this study was to clarify the improvement and sustainability of soil abiotic elements (nutrients) and the integration of biological elements (bacteria and fungi) with native habitat by *Medicago sativa*, artificial miscellaneous grass, *Rhus typhina*, *Pinus tabulaeformis*, *etc.*, which are widely used in vegetation restoration in northern China (Mou, Zhang & Liu, 2021; Song et al., 2021; Yin, Li & Du, 2021; Irene et al., 2022).

The Yanshan Mountains are not only a green barrier and water source of the capital Beijing China, but also a key area for ecological construction around Beijing. At the same time, Yanshan Mountains are also important mineral base in China. The eastern mining area in Sanhe city of Hebei Province is rich in dolomite, which lies at the southern foot of the Yanshan Mountains. It was once used as the supply base of construction raw materials from 1970s to 2015. An abandoned quarry with an area of 22 km^2^ had been formed because of decades of over exploitation. In our study, comparing to unrestored area and unmined area, we examined and compared the effects of herbaceous (including *Medicago sativa*, miscellaneous grass) and arboreal (including *Rhus Typhina*, orchard, *Pinus tabuliformis*) vegetation on soil characteristics and soil microbial community diversity and composition. This study also attempted to explore the following scientific issues: (1) Compared with unrestored plot, did vegetation restoration continuously improve soil nutrient status? Were there differences in soil nutrients under different vegetation restoration modes? (2) Compared with the original habitat plot, was there a trend of localization of soil microbial communities under different vegetation restoration modes? (3) If there were differences in soil microbial communities, which soil abiotic factors were related to them? The results were helpful to understand the response of reclaimed soil to vegetation restoration and provided reference for the selection of artificial vegetation.

## Materials and Methods

### Study area

Field experiments were carried out in the dolomite quarry (40°00′23″ N–40°02′34″ N, 117°05′13″ E–117°11′10″ E) in the east of the Yanshan piedmont plain. It has a typical warm temperate continental climate. The average annual temperature is 11.1 °C, the average annual precipitation is 617.4 mm, and the annual evaporation is 1,681.9 mm. The soil type in the study area is mainly calcareous saline-alkali brown soil, loose soil, poor erosion and erosion resistance, serious soil erosion, low content of organic matter. The original topsoil and vegetation of the quarry have been completely stripped, mostly new gravel conglomerate soil and sandy soil, lack of nutrients and water.

### Sample plot setting and experimental designing

In 2010, a large-scale artificial vegetation restoration measure was carried out in the quarry mining area by using native or non-native plant species, which mainly consisted of leguminous alfalfa (*Medicago sativa*) and miscellaneous grass (mainly Gramineae), and woody plants included *Rhus Typhina*, orchard (*Malus spp*., *Pyrus spp*., *Prunus spp*., *Crataegus pinnatifida*, *etc.*), *Pinus Tabuliformis*, *etc.* These species are more adaptable to the local fragile ecological environment and have a high survival rate, so they are often selected as pioneer species to improve the soil conditions (Table 1). In July 2020, seven sample plots of *Medicago sativa* (Me), miscellaneous grass (Mg), *Rhus Typhina* (Rh), orchard (Or), *Pinus tabuliformis* (Pi), unrestored plot (CK1) and unmined plot (CK2) were collected.

**Table 1:** The information of sample plots.

Sample plots	Experimental design and vegetation overview
CK1	CK1 was an unrestored plot. The site was a quarry platform formed in 2020, which had been covered with soil and leveled, but no artificial vegetation restoration had been carried out. Local annual herbaceous vegetation such as *Setaria viridis*, *Chenopodium album*, *Chloris virgata* and *Lepidium apetalum* were distributed. CK1 was taken as the starting point of vegetation restoration effect evaluation.
CK2	CK2 was an unmined plot. This sample plot was the natural habitat with less human disturbance. Natural vegetation included *Vitex negundo var. heterophylla*, *Elaeagnus angustifolia*, *Themeda triandra*, *Artemisia stechmanniana*, *Bothriochloa ischaemum*, *etc*. This sample plot was the evaluation standard of vegetation soil restoration effect.
Rh	Rh was a *Rhus Typhina* plot. This sample area was a pure *Rhus Typhina* forest, and there were few herbs under the forest. It had been restored for 10 years.
Mg	Mg was a miscellaneous grass plot. This sample plot was mainly gramineous plants such as *Eleusine indica*, *Zoysia japonica* and *Buchloe dactyloides*, mixed with *cosmos bipinnata* and *Lespedeza formosa*. It had been restored for 10 years.
Me	Me was a *Medicago sativa* plot. This sample plot had been artificially restored with herb (*Medicago sativa*) for 10 years. *Medicago sativa* declined, and local annual herbs such as *Setaria viridis*, *Salsola collina* and *Chenopodium album* appeared. It had been restored for 10 years.
Or	Or was an orchard plot. This sample plot was a variety of fruit trees, and there were a large number of natural weeds under the tree, including *Cymodon doctylon*, *Digitaria sanguinalis*, *Eleusine indica*, *Imperata cyindrica*, *Amaranthus lividus*, *Amaranthus retroflexus*, *Descurainia sophia*, *Humulus scandens* *etc*. It had been restored for 10 years.
Pi	Pi was a *Pinus Tabuliformis* plot. This sample plot was pure *Pinus tabulaeformis* forest, and there were few herbs under the forest. It had been restored for 10 years.

**Note:**

Among, CK1 was an unrestored plot. CK2 was an unmined plot. Rh was a *Rhus Typhina* plot. Mg was a miscellaneous grass plot. Me was a *Medicago sativa* plot. Or was an orchard plot. Pi was a *Pinus Tabuliformis* plot.

### Methods of soil investigation and measurement

The field work was carried out in August 2021, seven quadrats (10 m × 10 m) were set randomly according to the S-shape in the above four sample plots. On the diagonal of each quadrat, litters on the soil surface were removed, and 20 cm soil columns were randomly drilled with a 5 cm diameter soil drill. The above operation was repeated seven times for each sample plot. Finally, the seven soil samples of each plot were mixed adequately, and were sealed with a sterile plastic bag refrigerating in an ice box, and were quickly taken back to the laboratory. The soil samples were divided into two parts, one part (sieved through 2 mm mesh) was used to measure soil nutrients after natural air-drying in the room, and the other part (frozen at −20 °C) was used to measure soil microorganisms.

In this study, soil organic matter, nitrogen, phosphorus, potassium and pH were used as the indicators to characterize soil quality under different restoration modes. The corresponding methods are as follows, total nitrogen (TN): elemental analyzer (Euro Vector EA3000); total phosphorus (TP): spectrophotometer (UV-9000S) after digestion with H_2_SO_4_-HClO_4_.; total potassium (TK): sodium hydroxide melting method; pH: pH meter (Mettler Toledo pH (FE20)); organic matter (OM): potassium dichromate volumetric-external heating method; available nitrogen (AN): alkaline hydrolysis diffusion method; available phosphorus (AP): hydrochloric acid and sulfuric acid solution extraction method; available potassium (AK): ammonium acetate extraction-flame spectrophotometry. Seven repetitions were done per sample.

### Analysis of soil microbial community

Total DNA was extracted by CTAB assay and later measured by 1% agarose gel electrophoresis. V3+V4-b segment of bacterial 16S rRNA gene was amplified using 338F (5′-ACTCCTACGGAGCAGCA-3′) and 806R (5′-GGACTACHVGGGTWTC TAAT-3′) as primers (Xu et al., 2016). The ITS1-f segment of fungal 18S rRNA was sequenced using ITS1F (5′-CTTGGTCATTTAGAGGAAGTAA-3′) and 2043R (5′-GCTGCGTTCTTCATCGATGC-3′) as primers (Caban et al., 2018; Nottingham et al., 2018). The amplification conditions were as follows: initial deformation at 98 °C for 1 min, followed by 30 cycles at 98 °C for 10 s, 50 °C for 30 s, 72 °C for 60 s, and a final extension of 72 °C for 5 min (Applied Biosystems, Foster City, CA, USA), all purified PCR products were mixed in equimolar amounts by Qubit 2.0 Fluorometer (Thermo Fisher Scientific, Waltham, MA, USA). After DNA was extracted from the soil samples, they were sent to Biomarker Technologies Co, LTD, Beijing, China for high throughput sequencing of Illumina (Illumina, San Diego, CA, USA). Seven repetitions were done per sample.

### Statistical analyses

In this study, excel and R-3.6.3 Vegan Picante and other software were used for statistical analysis of all data. The Alpha (α) diversities of soil fungal and bacterial community were analyzed by QIIME2 (https://qiime2.org/). In order to analyze Beta (β) diversity of soil fungal and bacterial communities, firstly, the OTUs (Operational Taxonomic Units) table was standardized by Hellinger, Non-metric multidimensional scaling (NMDS) analysis of the phylogenetic of OTUs based on Bray-Curtis distance (Bray-Curtis Dissimilarity, d^BCD^) was calculated by using the Vegan package of R-3.6.3. Redundancy analysis was carried out using CANOCO for Windows, Version 4.5. VPA (variance partitioning canonical correspondence analysis). The samples were analyzed by using ANOSIM (Analysis of Similarities) and PerMANOVA (permutation-based multivariate ANOVA) variance analysis, so as to analyze whether there are significant differences among sites, and further analyze the differences among groups by PCoA (Principal Co-ordinates Analysis) (Edgar, 2013; Kõljalg et al., 2013).

## Results

### Soil physicochemical properties

**Figure 1:**
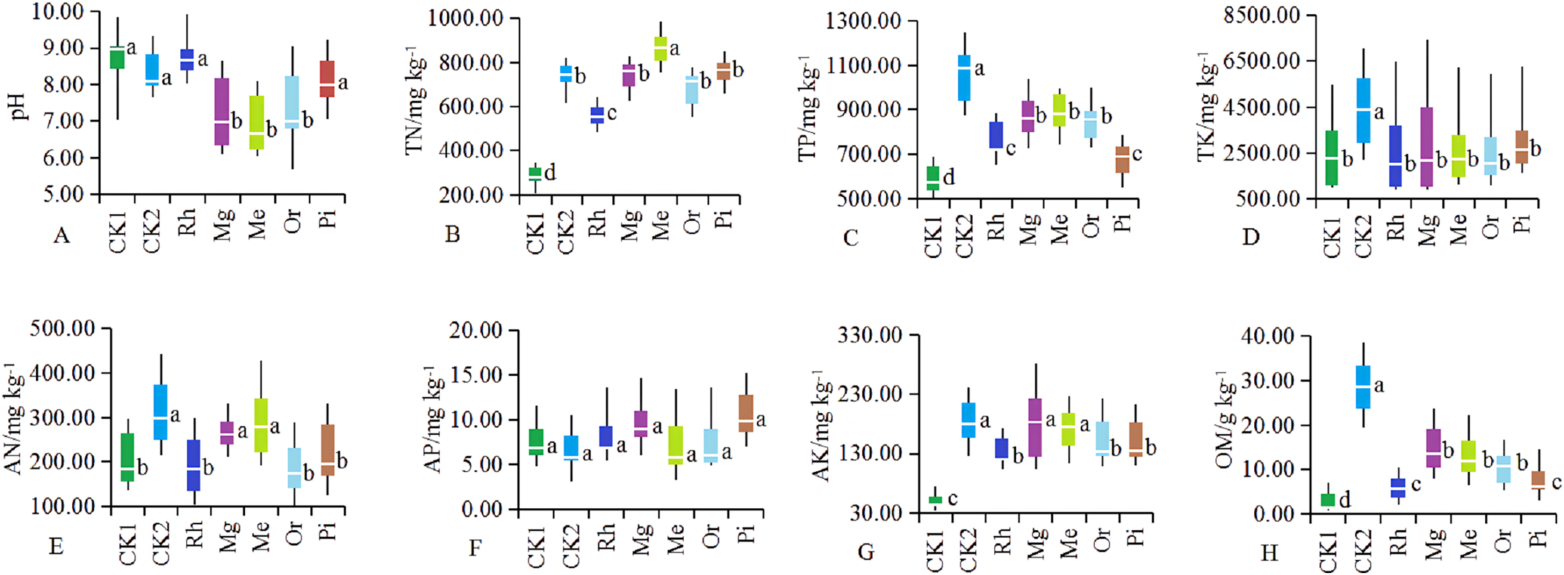
(A–H) Soil physicochemical properties in different sample plots. The different letters indicated significant differences between treatments according to ANOVA and Tukey’s test (*n* = 7, *P* < 0.05). The white line segments in each box indicated the average of the data group. The tops and bottoms of boxes represent the 75th and 25th percentiles, respectively. The upper and lower vertical bars extend to the maximum and minimum values, respectively.

Soil parameters such as pH, TN, TP, TK, AN, AP, AK and OM were significantly different in the different plots (Fig. 1). After vegetation restoration, the pH values of CK1 and CK2 were 8.89 and 8.22, while those of Me, Mg and Or were 6.59, 6.83 and 7.08, respectively (Fig. 1A). Obviously, herbaceous plots Me, Mg and woody plot Or could significantly improve alkaline soil, while the arboreal plots of Rh and Pi had no obvious effect on alkaline soil.

The concentrations of TN (Fig. 1B), TP (Fig. 1C), AK (Fig. 1G) and OM (Fig. 1H) in restored plots (Rh, Or, Pi, Mg and Me) showed significantly higher values compared with CK1 (*P* < 0.05), while TK (Fig. 1D) and AP (Fig. 1F) no significant differences were observed among restored plots (*P* > 0.05). In addition to the arboreal plots such as Rh, Or and Pi, herbaceous vegetation restoration also significantly increased the concentration of AN (Fig. 1E), with the most significant increase in the plots of Me and Mg. In general, the effect of Mg and Me on the improvement of soil nutrients were more prominent, followed by Or.

### Soil microbial properties

**Figure 2:**
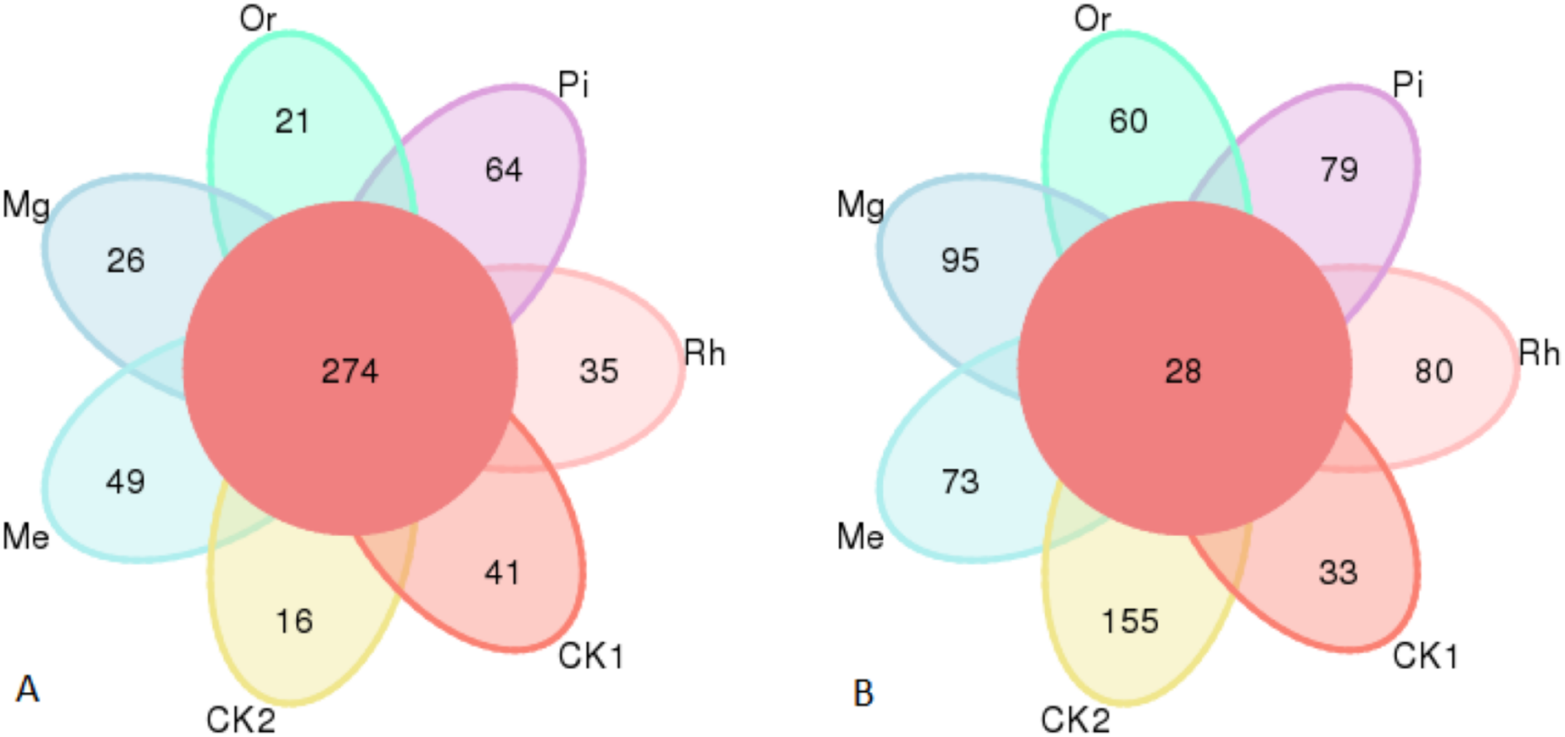
Venn diagram of soil bacterial OTUs (A) and fungal OTUs (B). The OTUs not unique to a single sample or common to all samples were not shown in the diagram.

Gene sequence analysis of bacteria and fungi in 49 samples provided a total of 460,556 and 538,732 effective sequences clustered into 2,381 OTUs and 1,288 OTUs at the similarity level of 97.0%, respectively, belonged to 32 phyla, 662 genera and 724 species for bacteria, 10 phyla, 298 genera and 326 species for fungi. The numbers of identified bacterial OTUs in the plots of CK1, CK2, Me, Mg, Or, Pi and Rh were 1,231, 1,684, 1,119, 1,633, 1,632, 1,222 and 878, respectively. Among them, there were 274 core OTUs in the seven groups, and soils of CK1, CK2, Me, Mg, Or, Pi and Rh had 41, 16, 49, 26, 21, 64 and 35 unique OTUs, respectively (Fig. 2A). Then, the fungal OTUs Venn diagram showed that there were 28 core OTUs in the seven groups, and the unique OTUs in the above plots were 33, 155, 73, 95, 60, 79 and 80, respectively (Fig. 2B).

**Figure 3:**
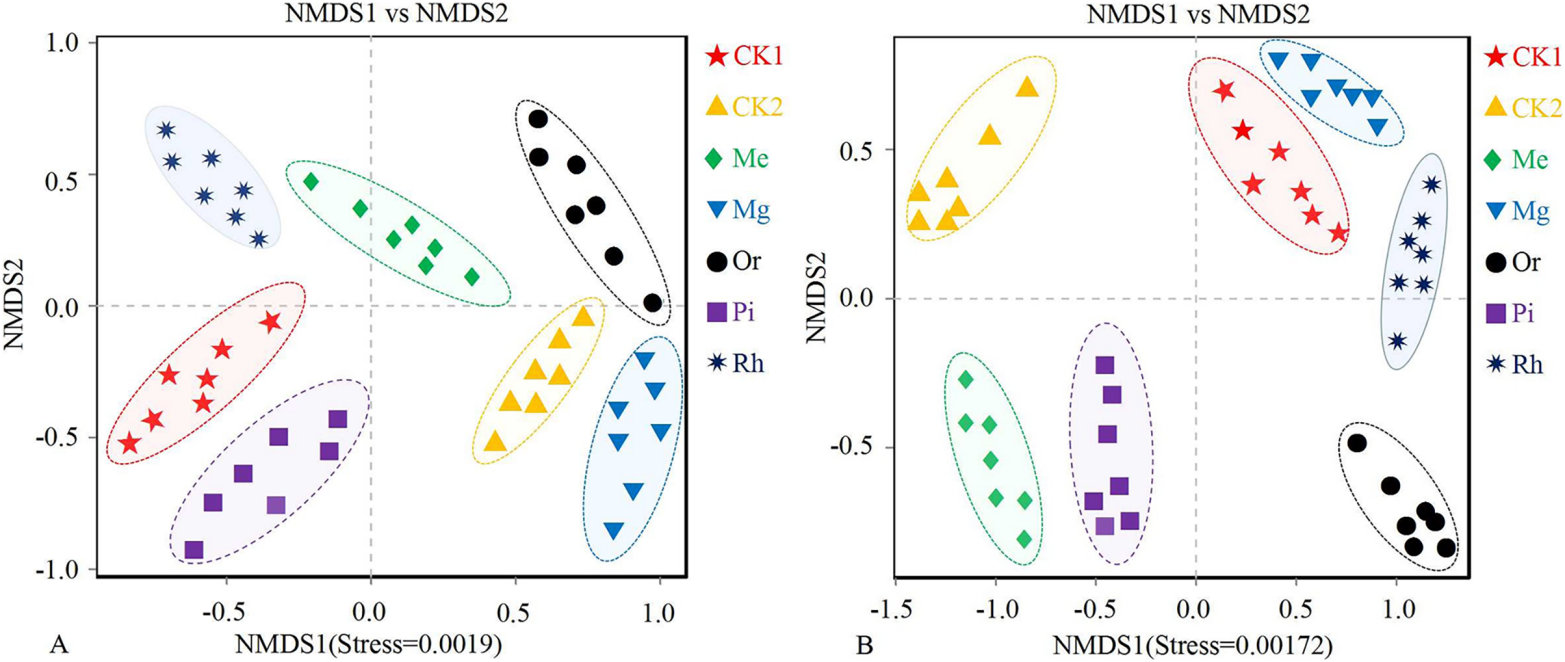
NMDS plots of the soil bacterial (A) and fungal (B) OTUs. Non-metric multidimensional scaling (NMDS) of the soil bacterial (A) and fungal (B) community composition (Bray–Curtis distance). Ellipses indicate 95% confidence intervals around centroids for each site.

NMDS analysis showed that no overlap was observed among different plots, which illustrated that soil bacterial (Fig. 3A) communities (stress = 0.0019) and fungal (Fig. 3B) communities (stress = 0.00172) in plots were clearly different, especially the bacterial (Fig. 3A) composition between CK1 and restored plots (Mg and Or), and the fungal (Fig. 3B) composition between CK1 and all plots except Mg.

Furthermore, the cluster dendrogram (Fig. S1) based on Unweighted Pair-group Method with Arithmetic Mean (UPGMA) showed that the soil bacterial communities in CK1 were far from that in Mg and Or, which were close to that of the CK2, Mg and Or (Fig. S1A). It showed that vegetation restoration could change the soil bacterial community to varying degrees. The results demonstrated that among various restoration modes, the localization trend of soil bacteria in the Or and Mg were more obvious. The variation law inconsistent with that of bacteria was found in the soil fungal community. Seven samples were divided into four clusters (Fig. 3B) by cluster analysis (each cluster was almost located in different quadrants). The larger cluster group were from the plots of CK1, Mg and Rh plots (Fig. S1B), indicating that the modes of Me, Or and Pi could significantly change the soil fungal community compared with Mg and Rh. The sample grouping of CK2 plot was clearly separated from other plots. Obviously, at this stage, the soil fungal communities in each restoration mode had not shown the trend of localization.

**Figure 4:**
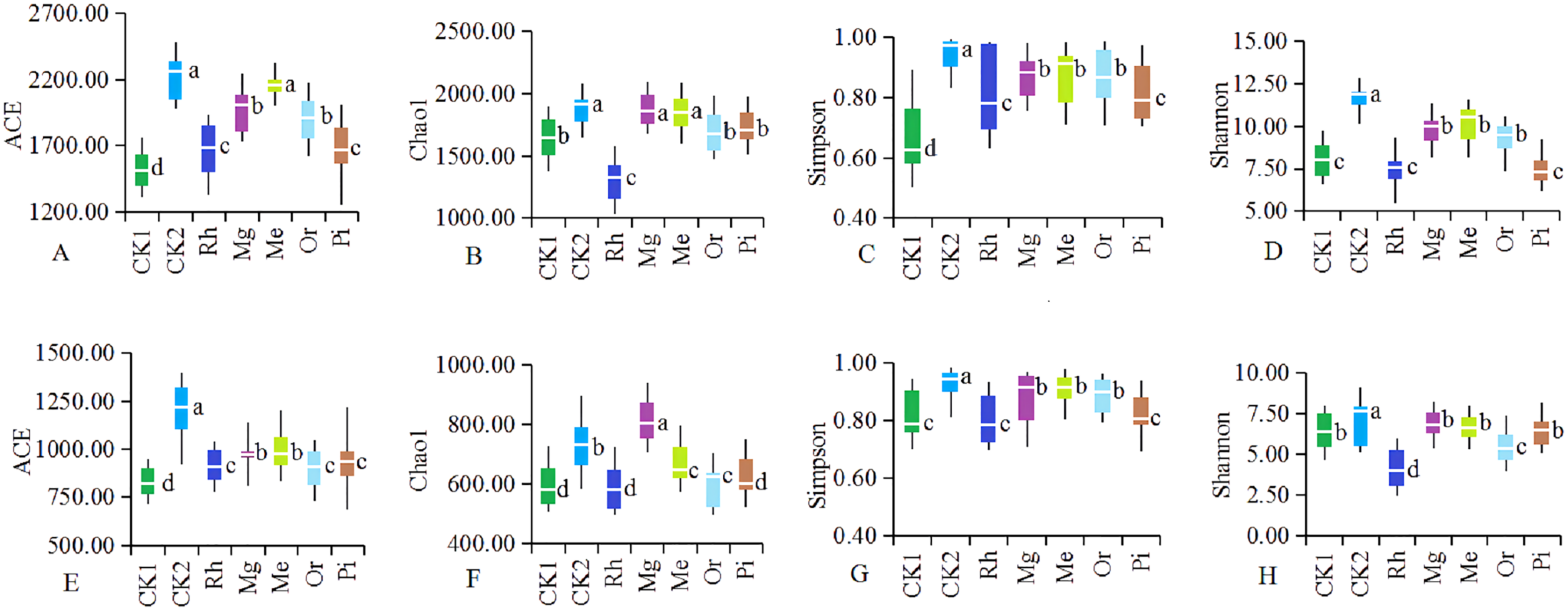
Diversity of the soil bacteria (A–D) and fungi (E–H). Alpha diversity of the soil bacteria (A–D) and fungi (E–H). The white horizontal bars inside each box indicates the mean value. The tops and bottoms of boxes represent the 75th and 25th percentiles, respectively. The upper and lower vertical bars extend to the maximum and minimum values, respectively. The numbers of replicated samples in this figure are *n* = 7. Letters are used to distinguish whether there are significant differences between groups. Different letters indicate that there are display differences between groups (*P* < 0.05).

As the number of sequences per sample increased, the Rarefaction Curve of OTUs (Fig. S2) reached the plateau. Re-vegetation could significantly increase soil bacterial community diversity as shown in Fig. 4. The unrestored plot CK1 exhibited the lowest values of ACE index (Fig. 4A1) and Simpson index (Fig. 4A3). The highest values of bacterial ACE index and Simpson index were observed in the herbaceous plot Me, followed by the herbaceous plot Mg and the woody plot Or respectively. Woody plot Rh exhibited the lowest values of Chao1 index (Fig. 4A2) and Shannon index (Fig. 4A4). While Me owned the highest values of bacterial Chao1 index and Shannon index with 1,873.28 and 10.84. With regard to soil fungal community diversity (Figs. 4B1– 4B4), showing a change law almost consistent with that of bacteria: the fungal diversity indexes of the natural habitat plot CK2, the herbaceous plots Me and Mg were higher, followed by the woody plots Or and Pi, and the unrestored plot CK1 and the woody plot Rh were the lowest. Thus, in this study area, herbaceous vegetation restoration modes of Me and Mg better improved the diversity of soil microorganism, while woody modes of Or was better, but Rh was the worst.

**Figure 5:**
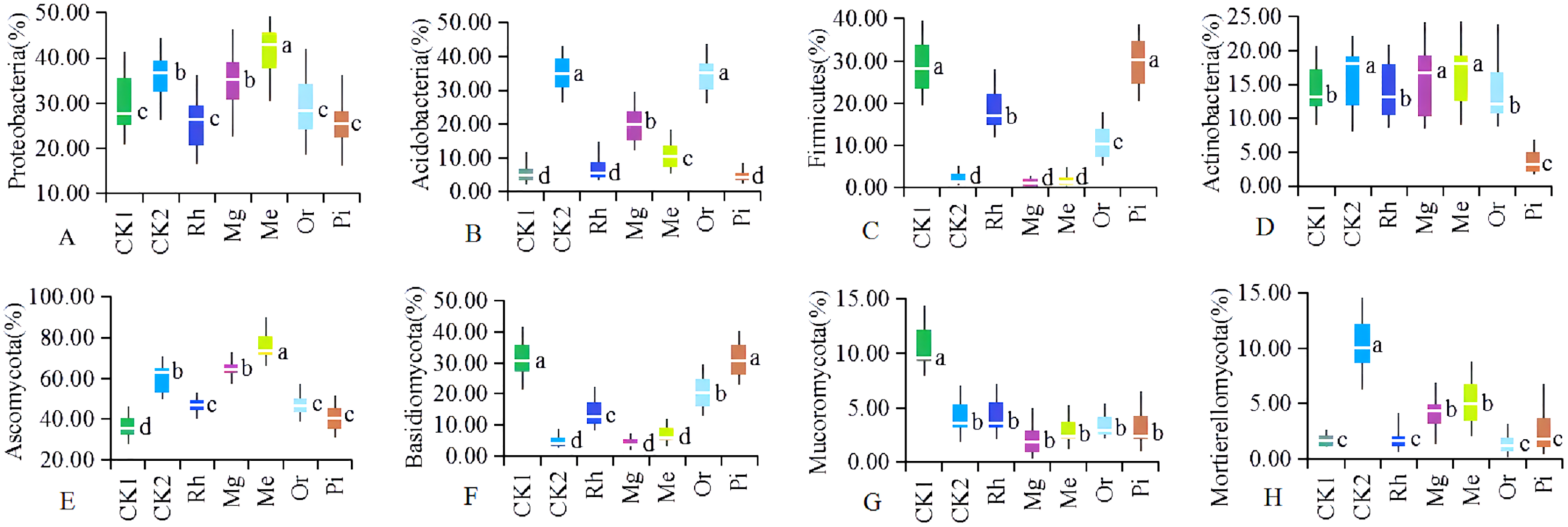
The relative abundances of dominant soil bacterial (A–D) and fungal (E–H). Communities at the phylum level. Letters are used to distinguish whether there are significant differences between groups. Different letters indicate that there are display differences between groups (*P* < 0.05, ANOVA).

In studied plots, the OTUs could be divided into different categories, and four dominant bacterial phyla with the average relative abundances >12% were obtained, including Proteobacteria (Fig. 5A1), Acidobacteria (Fig. 5A2), Firmicutes (Fig. 5A3) and Actinobacteria (Fig. 5A4), accounting for 67.84–76.49% of the total relative abundances. Among them, Proteobacteria and Acidobacteria had absolute advantages, accounting for 31.49% and 15.99% of the average relative abundance, respectively.

It could be seen from Fig. 5A1 that the relative abundances of Proteobacteria in the plots of Mg, Me and CK2 were significantly higher than that of CK1, and that of plot Me were significantly highest; The relative abundance of Proteobacteria were no significant difference between Mg and CK2; There was no significant difference in the relative abundance of Proteobacteria among Rh, Or, Pi and CK1. The relative abundances of Acidobacteria (Fig. 5A2) in plots CK2 and Or were significantly higher than that of all other plots, and which in Me and Mg were significantly higher than that of CK1, Rh and Pi. Obviously, compared with plot CK1, plots Or, Me and Mg significantly increased the abundance of Acidobacteria. In the studied area, the bacteria of Firmicutes (Fig. 5A3) were mainly distributed in plot CK1 and the arbor forest plots such as Rh, or and Pi. In summary, the relative abundances of Actinobacteria (Fig. 5A4) in herbaceous plots Me, Mg and natural habitat plot CK2 were significantly higher than that of unrestored plot CK1 and arbor forest plots such as Rh, Or and Pi.

Soil fungal phyla with the average relative abundances >3% were Ascomycota (Fig. 5B1), Basidiomycota (Fig. 5B2), Mucoromycota (Fig. 5B3) and Mortierellomycota (Fig. 5B4), accounting for 63.25–90.68% of the total relative abundance. Among them, Ascomycota and Basidiomycota were absolutely dominant, accounting for 53.19% and 17.45% of the average relative abundance, respectively.

In the herbaceous plots Me and Mg, the relative abundances of Ascomycota (Fig. 5B1) were significantly higher than that of other restored plots and the unrestored plot CK1; The relative abundances of Ascomycota in the arboreal plots of Rh, Or and Pi were lower and had no significant difference, but they were significantly higher than that of CK1 and significantly lower than CK2 in the original habitat plots. The relative abundance of Basidiomycota (Fig. 5B2) showed the opposite change law to that of Ascomycota, that was, the relative abundances of Basidiomycota in arboreal plots were significantly higher than that in herbaceous plots and CK2. Vegetation restoration significantly reduced the abundance of Mucoromycota. The relative abundance of Mucoromycota (Fig. 5B3) in plot CK1 was significantly higher than that in all other plots, while there was no significant difference in the relative abundance of Mucoromycota among other sample plots (including CK2). The relative abundance of Mortierellomycota (Fig. 5B4) showed the same variation law as that of Ascomycota, which were significantly higher in herbaceous plots Me and Mg than that in unrestored plot CK1 and arboreal Rh, Or and Pi.

**Figure 6:**
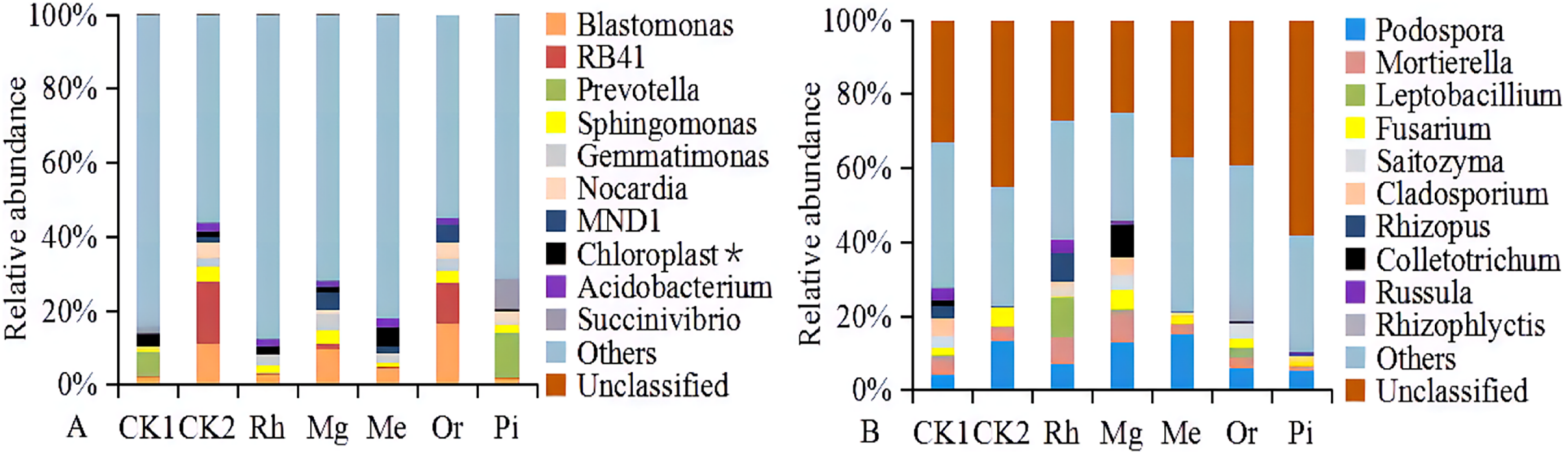
The relative abundances of soil bacterial (A) and fungal communities (B). With the relative abundance >1% at the genus level.

At the genus level, the bacterial groups with the average relative abundances greater than 1.0% were *Blastomonas*, *RB41*, *Prevotella*, *Sphingomonas*, *Gemmatimonas*, *Nocardia*, *MND1*, uncultured_bacterium_o_Chloroplast, *Acidobacterium* and *Succinivibrio*, accounting for 12.21% (Rh)–45.27% (Or) in different plots (Fig. 6A). In addition, the fungal groups with the relative abundances greater than 1.0% were *Podospora*, *Mortierella*, *Leptobacillium*, *Fusarium*, *Saitozyma*, *Cladosporium*, *Rhizopus*, *Colletotrichum*, *Russula* and *Rhizophlyctis*, accounting for 11.06% (Pi)–47.18% (Mg) in different plots (Fig. 6B). The heatmap (Fig. S3) indicated that the soil bacterial community structure of seven plots could be divided into five relatively concentrated clusters, including CK2-Or-Mg, CK1, Rh, Pi and Me, the results showed that the soil bacterial community could be changed by vegetation restoration (Fig. S3A). Among them, soil bacterial community composition from Mg and Or existed much more similar in comparison to CK2, demonstrating that the plots Mg and Or could better improve the soil bacterial community structure compared to CK2 (Fig. S3A). While, soil fungal community structure were clustered into five groups, including Rh-Mg-CK1, Me, Or, Pi and CK2 (Fig. S3B). The similarities of soil fungal community composition of Mg, me, Rh, Or and Pi were very low compared with the natural habitat plot CK2. While, a certain similarity was reflected between Mg and the unrecovered plot CK1, demonstrating that all recovered plots could not better improve the soil fungal community structure compared to CK2 (Fig. S3B).

### Correlation between soil abiotic and biotic characteristics

The correlations between soil characteristics and microbial community diversity were shown in Table 2. The concentrations of soil TN, TP, AN, AK and Om had a strong positive effect on soil bacterial and fungal diversity. The soil microbial diversity indexes of Simpson, Chao1 and ACE increased with the decrease of Soil pH.

**Table 2:** Spearman’s rank correlations between soil characteristics and soil microbial communities.

Microbial community	Diversity index	Soil characteristics							
		pH	TN	TP	TK	AN	AP	AK	OM
Bacteria	Simpson	−0.53*	0.54*	0.51*	0.31	0.38	0.39	0.69**	0.37
	Chao1	−0.68**	0.67**	0.43	0.53*	0.58*	0.22	0.49	0.67**
	ACE	−0.58**	0.67**	0.41	0.26	0.65**	0.12	0.51*	0.65**
	Shannon	−0.41	0.76**	0.60*	0.21	0.54*	0.36	0.59**	0.63**
Fungi	Simpson	−0.51*	0.21	0.31	0.18	0.32	0.21	0.23	0.52*
	Chao1	−0.77**	0.77**	0.59*	0.33	0.59*	0.33	0.65**	0.69**
	ACE	−0.63**	0.68**	0.56*	0.36	0.66**	0.41	0.71**	0.66**
	Shannon	−0.29	0.47	0.27	0.28	0.55*	0.39	0.43	0.55*

**Notes:**

*n* = 7. Significance are demonstrated as: *P* < 0.05 (*) (two tailed), *P* < 0.01 (**) (two tailed).

Soil TN, TP, AN, AK and OM concentrations strongly positively influenced soil bacterial and fungal community diversity. However, the diversity indexes of soil microorganisms, such as Simpson, Chao 1 and Ace, increased with the decrease of soil pH.

**Figure 7:**
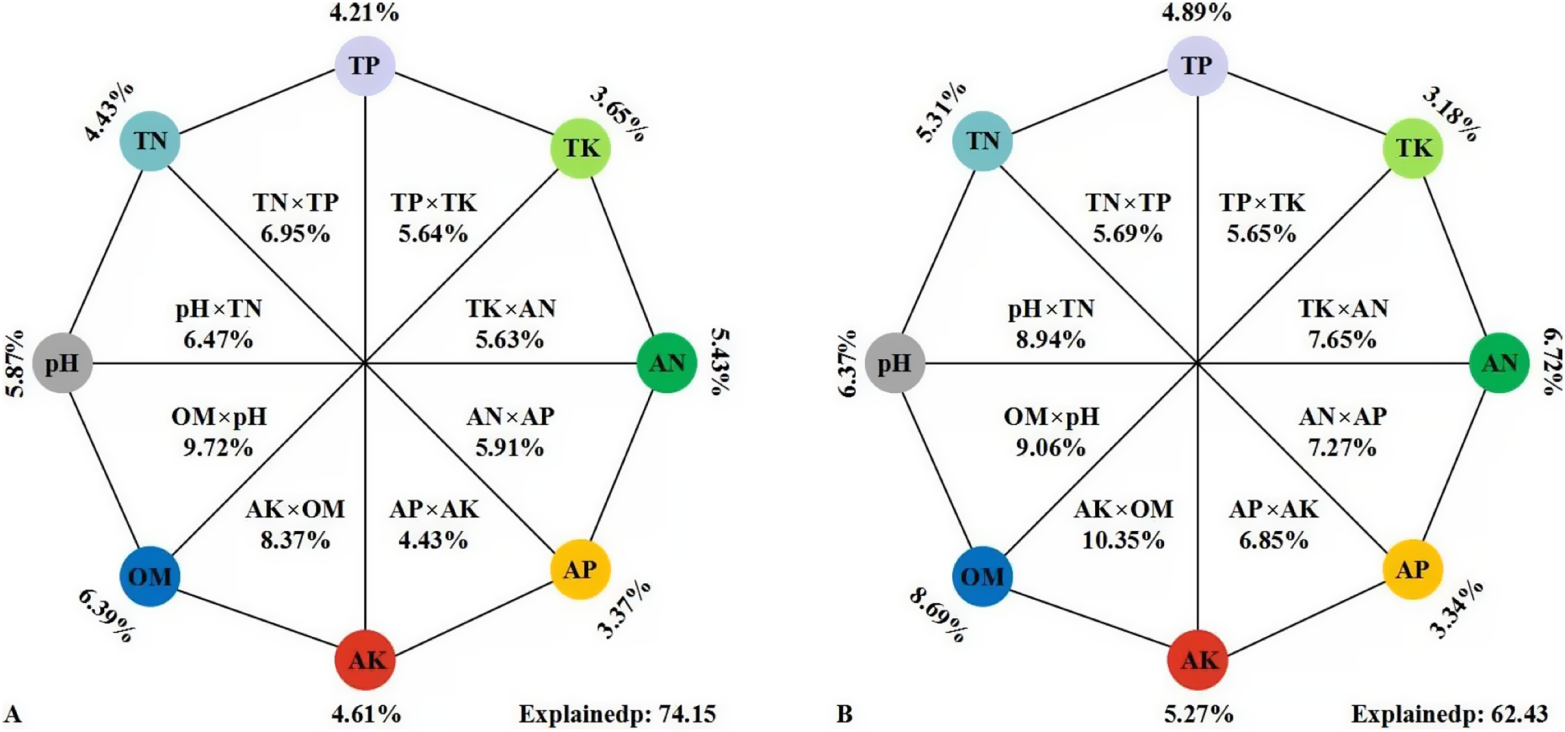
VPA of the effects of soil environment factors on soil bacterial (A) and fungal (B) communities. The percentages in the figures were the variation of the bacterial/fungal community structure explained by the eight sets of environmental factors. The external percentages were the single interpretation rate of environmental factors, the internal percentages were the common interpretation rate of two environmental factors, and the relative abundances of OTUs were used as input in the analysis.

A variety of soil environmental factors act on soil microbial communities. In this study, VPA (Fig. 7) was conducted to quantify the relative contribution of different environmental factors to changes in microbial community composition. These eight environmental variables accounted for 74.15% of the changes in soil bacterial community. The alone relative contribution of soil properties to soil bacterial community structure showed a downward trend: OM (6.39%), pH (5.87%), AN (5.43%), AK (4.61%), TN (4.43%), TP (4.21%), TK (3.65%) and AP (3.37%) of the total variation, respectively (Fig. 7A). These variables explained 62.43% of the variation of soil fungi. The effect of soil properties on soil fungal community structure decreased in turn of OM (8.69%) > AN (6.72%) > pH (6.37%) > TN (5.31%) > AK (5.27%) > TP (4.89%) > AP (3.34%) > TK (3.18%) (Fig. 7B). The results showed that soil OM was the most important factor, which was highest as a single factor for explaining the variation, followed by pH and AN, while soil TK and AP were the least important factors.

## Discussion

### Soil physicochemical properties under different modes

Soil quality is a significant component of ecosystem restoration, and its physical, chemical and biological properties sustain plant regeneration and establishment (Yu et al., 2020). After mining activities, soil had been destroyed severely with a higher pH, a lower soil nutrients (Fig. 1). The results in this study showed that vegetation restoration could generally improve the soil quality (Fig. 1). One common reason was that in the process of vegetation restoration, the gradual accumulation of surface litter increases the humus in the soil, so as to effectively improve the soil nutrient status (Li, Liu & Zhou, 2015; Li et al., 2018). And there were significant differences in the effects of different restoration modes on different soil nutrients.

*Medicago sativa* is a widely distributed artificial vegetation type in northern China, with strong alkali resistance, especially in accelerating vegetation restoration (Franchini et al., 2005), preventing soil erosion (Wu et al., 2013a), repairing degraded soil and so on (He et al., 2017). A large number of rhizobia are formed in alfalfa roots, which have the function of nitrogen fixation. Mg was a miscellaneous grass plot, which was mainly gramineous plants such as *Eleusine indica*, *Zoysia japonica* and *Buchloe dactyloides*, mixed with *cosmos bipinnata* and *Lespedeza formosa* (Table 1). In the plots Me and Mg, a large number of roots and litter rotted into the soil, which significantly increased the content of soil nutrients (Figs. 1B, 1C, 1E, 1G and 1H) and improved the alkaline soil (Fig. 1A). The research of Guo et al. (2009) shows that the soil pH value in the restored area is significantly lower than that in the unrestored area, which may be caused by the decay of plant residues and roots (Mukhopadhyay, Maiti & Masto, 2013).

In the ecological restoration, vegetation and soil are coordinated restoration. Restoration of plant diversity in nutrient-poor soil leads to an increase in soil fertility (George & David, 2019). Moreover, the modes of Me and Mg also help to improve the biodiversity of plant communities. In the process of vegetation restoration in northern China, the decline and localization of alfalfa (Li & Shao, 2005; Chen & Li, 2015) and artificial miscellaneous grass (Niu & Jiang, 2004; Pan et al., 2020) are inevitable trends. At present, there were a large number of native dominant herbs such as *Bothriochloa ischaemum*, *Themeda triandra*, *Melilotus albus* in the plots Me and Mg (Table 1). There was no doubt that Me and Mg greatly improved the nutritional status of the soil, and then promoted the emergence (settlement) of native plants, which in turn promoted the localization of soil microorganisms (Fig. 3A) (Faivre et al., 2017; He et al., 2020).

In the studied area, the modes of *Rhus typhina* and *Pinus tabulaeformis* had been used on a large scale. *Rhus typhina* has a wide ecological range and strong ability of reproduction and stress resistance, which plays an important role in vegetation restoration in arid and mining areas. Due to the characteristics of *Rhus typhina*, some scholars believe that it has the characteristics of invasive species and may develop into invasive species (Liu, Yu & Zhou, 2002; Wang et al., 2012), which poses an ecological threat to native species.

Although *Rhus typhina* can rapidly increase vegetation coverage and biomass in the short term. However, in the long run, with the rapid propagation of *Rhus typhina*, the herbs under the forest decrease sharply, forming a single excellent community of *Rhus typhina*, but unable to form a stable local plant community (You et al., 2013). This study showed that in the plot Rh, because the *Rhus typhina* had the characteristics of super root tiller reproductive ability and stress resistance, the native plants were difficult to grow (Table 1).

*Pinus tabulaeformis* is an important greening tree species in northern China, but in the process of its long-term management, it is often found that there are problems such as productivity decline, soil fertility decline and renewal obstacles (Cao et al., 2015). *Pinus tabulaeformis* has a strong autotoxic effect, which is caused by the leaching of its surface stems and leaves through rain and fog droplets or the generation of odor substances (Li, Wang & Yao, 2010). The Autotoxicity of *Pinus tabulaeformis* not only limits the growth and regeneration of its own seedlings, but also hinders the entry of other native species (Wang et al., 2007; Pan et al., 2009).

In the studied area, as an attempt of ecological restoration of mining wasteland, many fruit orchards had been planted because of their economic value. Compared with the modes of Rh and Pi, the allelopathy of Or mode is not obvious (Wu et al., 2013b). Therefore, there were also a large number of naturally occurring weeds (Table 1) in the plot Or, and previous studies (Hao et al., 2003; Li, Zhao & Zhang, 2004) have shown that planting grass in orchard can increase the content of soil OM (Fig. 1H), TN (Fig. 1B) and TP (Fig. 1C), which was conducive to the absorption of nutrient elements by fruits. The soil pH in this study area was generally high and belonged to weakly alkaline soil. Alkaline soil can cause physiological disturbance and element deficiency in fruit trees (Guo et al., 2015).

Moreover, according to this study, it should also be pointed out that some soil nutrients were not related to the restoration modes, that was, compared with the non restored plot CK1, there were no significant difference of the soil TK (Fig. 1D) and AP (Fig. 1F) between the restoration modes. The concentration of soil AP in the study area was generally low, all below 10 mg . kg^−1^, and the consumption of soil AP was very small (Guo et al., 2009). Another study (Qian et al., 2014) shows that the weak mobility and low utilization of AP in alkaline soils (Fig. 1F) resulted in an overall low accumulation of TP (relative to CK2) (Fig. 1C). The TK level in all soils was significantly lower than that of natural habitat soils, which may reflect that each restoration mode had no obvious effect on the accumulation of total potassium to a certain extent (Liu et al., 2004). This may be related to the low concentration of potassium in saline alkali soil in the study area, which mainly exists in the form of mineral potassium and non exchangeable potassium, and the low concentration of exchangeable potassium and water-soluble potassium.

To sum up, compared with woody modes (Rh, Pi and Or), herbaceous modes (Me, Mg) reduced topsoil erosion, increased litter, and improved soil environment and plant community, creating a good environment for the growth of more native plants and soil microorganisms.

### Soil microorganisms under different modes

Soil microbial community diversity indexes changed with different vegetation restoration modes and exhibited higher in some restored plots compared with the unrestored plot CK1 (Fig. 4). On the whole, the herbaceous modes were better than the woody modes. Previous studies have shown that the establishment of rich plant communities promotes the development of a variety of microorganisms. Soil microbial community composition changed with different restored plants. The NMDS (Fig. 3) showed that the composition of bacterial and fungal communities were different between all plots, indicating the existence of different soil microbial ecosystems, similar results obtained in previous studies (Kim et al., 2018).

At the phylum level, the bacterial populations of Proteobacteria (Fig. 5A1), Acidobacteria (Fig. 5A2), Firmicutes (Fig. 5A3) and Actinobacteria (Fig. 5A4) were dominant, indicating that these microbial communities had high adaptability and played a vital role in these ecosystems.

Proteobacteria, which are the main dominant community in alkaline soil and widely exist in mining area (Li, Liu & Zhou, 2015), are widely distributed in different environments and have strong adaptability (Liu, Huang & Zeng, 2016). Proteobacteria are often the dominant taxa in some black soil and semi humid soil, the larger the proportion of Proteobacteria in the soil, to some extent, it represents the more fertile the soil (Liu et al., 2014). This study showed that compared with CK1, the relative abundance of Proteobacteria (Fig. 5A1) increased in other sample plots, which showed the same trend as the change law of bacterial community composition in the natural succession of grassland vegetation on the Loess Plateau (Zhang et al., 2016). This study also confirmed that *Medicago sativa* (Me) and artificial miscellaneous grass (Mg) could significantly improve the content of Proteobacteria. However, woody species such as *Rhus typhina* (Rh), orchard (Or) and *Pinus tabulaeformis* (Pi) did not show obvious effect. The similar findings in zinc smelting slag (Luo et al., 2015), as well as copper mine tailing in Jiangxi Provience demonstrated that the predominant phylum was Proteobacteria (Sun et al., 2018).

Acidobacteria are also very common in the natural environment and can degrade macromolecular polymers such as plant cellulose (Lv, Zhao & Zhang, 2020). The abundance of Acidobacteria in some forest soil bacteria is more than 65% (Lee, Ka & Cho, 2008). This study showed that Acidobacteria (Fig. 5A2) mainly existed in the plot Or, secondly, followed by the plots Mg and Me, which were related to a large number of soil OM (Fig. 1H) and soil nutrients (Fig. 1B, 1C, 1E and 1G) in the plots Or, Mg and Me. Additionally, previous report in restored mine soils also established that phytoremediation of leguminous and gramineous herbs could significantly increase the relative abundance of Acidobacteria (Wei et al., 2018).

In arid and oligotrophic soils, the relative abundance of Firmicutes is high (Vanessa et al., 2013). The cell wall of Firmicutes is thick, which can produce spores and resist dehydration, so it can better adapt to the harsh environment in the mining area (Ji et al., 2014). In the plots CK1, Rh, Or and Pi, there were almost no local herbaceous vegetation cover, and the soil moisture content was low, so the relative abundance of Firmicutes (Fig. 5A3) was high. In the plots Me and Mg, the vegetation cover increased significantly and the soil water content increased, so the relative abundance of Firmicutes showed a downward trend. The relative abundance of Firmicutes in the plot CK2 was the lowest, which was related to soil water content under different restoration modes (Yu et al., 2020).

Actinomycetes play an active role in the material cycle, promote the formation of soil aggregate structure and improve the soil (Johnson et al., 2007). As a marker phylum of soil nutrients, Actinomycetes have higher abundance in the weak acidity, arid and organic matter rich soils, and exhibits significant resilience to thrive in hostile environments (Stevenson & Hallsworth, 2014). Just like Proteobacteria and Acidobacteria, the relative abundance of Actinomycetes (Fig. 5A4) in the plots Me and Mg, where soils were rich in organic matter and soil nutrients, were significantly higher (relative to the unrestored plot CK1 and the arboreal plots Rh, Pi and Or), which was similar with the research in the gold mine tails (Sibanda et al., 2019).

Ascomycota and Basidiomycota are the two most widely distributed and abundant fungal groups, which are related to their metabolic characteristics and strong viability in a variety of habitats (Al-Sadi et al., 2017). In the studied area, the relative abundances of Ascomycota (Fig. 5B1) and Basidiomycota (Fig. 5B2) were high, which were similar to the results of Yang et al. (2017) in returning farmland to grassland in the semi-arid area of the Loess Plateau. Most Ascomycota and Basidiomycota are saprophytic bacteria. In this study, the pH values of soil in various plots were 6.9–8.9 (Fig. 1A), and neutral and alkaline soil is most suitable for the growth of saprophytic fungi, which may be one reason why Ascomycota and Basidiomycota are dominant bacteria (Zhang et al., 2018a, 2018b).

Ascomycota are susceptible to plant species and plant residues, and their function is to decompose lignified debris (De Boer et al., 2005). In this study, the soil nutrients, vegetation coverage and vegetation residues in alfalfa plot Me and artificial miscellaneous grass plot Mg increased significantly, so that Ascomycota can make better use of degradable vegetation residues and promote the rapid growth and reproduction of Ascomycota (Ma et al., 2013). Mortierellomycota is a marker group of rich soil nutrients. The relative abundance of Mortierellomycota (Fig. 5B4) in various plots presented the same change law as Ascomycota (Fig. 5B1), which is closely related to soil nutrients (Yuan et al., 2020).

More than 98% of terrestrial fungi in nature belong to Ascomycota and Basidiomycota, and the species diversity of the former is significantly more than that of the latter. The number of species of Ascomycetes is more than twice that of Basidiomycota (Hibbett et al., 2007). The relative abundance of Basidiomycota (Fig. 5B2) showed the opposite change law to that of Ascomycota (Fig. 5B1), which is related to the increase of fungal dominance of Ascomycota (Tian et al., 2017).

The spore germination and mycelial growth of Mucoromycota are fast (Lü et al., 2019). Therefore, dominant fungi are first formed in the early stage of vegetation restoration. However, Mucoromycota fungi are more sensitive to the accumulation of their own metabolic by-products, especially the accumulation of CO_2_ in the environment, which makes them stop growing, produce dormant structure and enter dormant state (Fröhlich-Nowoisky et al., 2009; Amend et al., 2010). In this study, the CK1 was a sample plot without artificial restoration, and only local annual herbaceous vegetation such as *Setaria viridis*, *Chenopodium album*, *Chloris virgata* and *Lepidium apetalum* were distributed. The relative abundance of Mucoromycota (Fig. 5B3) in sample plot CK1 was the highest. With the increase of recovery years, the relative abundance of Mucoromycota in all other plots decreased with the consumption of available nutrients and the accumulation of CO_2_ in soil, and which were no significant difference among various plots.

From the analysis of NMDS (Fig. 3), we could also obtain the following information: Compared with the original habitat (CK2), the recovery progress of soil bacteria and fungi was different in different restoration modes. The localization trend of soil bacteria in herbaceous plots was obvious, that was, the bacterial composition of Mg and Me was similar to that of CK2. However, in woody plots, only soil bacteria in Or showed an obvious localization trend. The similarity of fungal components between each model and CK2 was very low. Obviously, the influence of herbaceous vegetation on bacteria was more obvious at present, which was related to factors such as soil cover (saline-alkaline soil), recovery time (10 years, relatively short) and natural environment (more rainfall). The pH values of soils in the study area ranged from 7.5 to 8.5. Some studies have shown that the conditions of saline-alkali land are not conducive to the growth of fungi, and the bacterial diversity of microorganisms in saline-alkali land often has absolute advantages (Zhang & Feng, 2008; Zhou et al., 2006). There was more rainfall in the study area, and plants provided more carbon sources for soil bacterial communities. Thus, bacteria are resilient (de Franciska et al., 2018), so in this study, the recovery process was faster for bacteria, whereas fungi were less competitive (Harris, 2003). Zhang et al. (2021) shows that even after nearly 30 years, the soil quality after reconstruction was still difficult to return to the same level as that of ordinary agricultural land. This study also fully showed that soil nutrient status could be effectively regulated by artificial methods, but the localization of soil microorganisms was long and uncertain (Singh, Pandey & Singh, 2011). Therefore, from the perspective of soil microbial recovery, ecological restoration in the study area was still at an early stage (Bai et al., 2018; Zhang et al., 2021).

### Factors impacting on soil microbial communities

In each vegetation restoration mode, the significant abiotic changes were the accumulation of TN, TP, AN, AK and OM, which strongly affect the diversity of soil bacterial and fungal community (Table 2). These results were similar to previous findings (Bradley, Drijber & Knops, 2006; Griffiths et al., 2011).

In this study, the indexes of Simpson, Chao1 and ACE of bacteria and fungi were significantly negatively correlated with soil pH, which was inconsistent with the positive correlation between soil fungal richness and pH of non-alkaline soil reported in previous research (Liu et al., 2018), which was obviously related to saline-alkaline soil in the study area (Zhang & Feng, 2008). Our results were consistent with those of previous studies, suggesting that soil pH was a major factor controlling bacterial and fungal community structure (Yang et al., 2018; Rousk et al., 2010; Li et al., 2021).

In addition, other investigations have shown that TN, TP and AN in soil have effect on the composition of bacterial communities (Cho, Kim & Lee, 2016; Dawud et al., 2016; Zeng, Dong & An, 2016). Together, these findings support the idea that soil microorganisms are closely related to soil nutrients induced by different vegetation (Siles & Margesin, 2016). Our results revealed the effects of artificial vegetation and soil nutrients on soil microbial diversity and composition, and provided guidance for the artificial regulation of soil microbial diversity and composition in the restoration of abandoned mining areas.

## Conclusions

This study mainly analyzed soil nutrients and microorganisms under different vegetation restoration modes from two aspects: the improvement of physical and chemical properties and the localization process of soil ecosystem based on soil microbial community analysis. First, we had found that the herbaceous vegetation of *Medicago sativa* and artificial miscellaneous grass were more suitable for rapid and efficient soil restoration in Yanshan Mountains of Hebei Province, China. After more than 10 years of continuous cultivation, most of the soil physical and chemical properties had been improved. And the arboreal modes of *Rhus typhina* and *Pinus tabuliformis* had no significant effect on soil improvement. Moreover, the herbaceous vegetation restoration modes significantly increased the relative abundance of Proteobacteria, Acidobacteria, Actinobacteria, Ascomycota and Mortierllomycota. This study also revealed that the trend of bacterial localization in the fruit orchard, artificial miscellaneous grass and *Medicago sativa* soil was more obvious. Among many soil abiotic factors, OM, AN and pH were the most important factors affecting soil microbial community.

## Supplemental Information

10.7717/peerj.14359/supp-1 Supplemental Information 1 Bacterial (A) and fungal (B) UPGMA clustering tree based on Bray-Curtis algorithm.

10.7717/peerj.14359/supp-2 Supplemental Information 2 Rarefaction curves of soil bacterial (A) and fungal (B) communities. Rarefaction curves about the OTUs reached the plateau with the increase of the number of sequences per sample, stressing that the 35,000 and 20,000 sequences for each sample were sufficient to characterize soil bacterial and fungal communities in the studied soils.

10.7717/peerj.14359/supp-3 Supplemental Information 3 The heatmap of soil bacterial (A) and fungal (B) communities. The heatmap based on bray distance indicated that the soil bacterial community structure of seven plots could be divided into five relatively concentrated clusters.

10.7717/peerj.14359/supp-4 Supplemental Information 4 The statistics of bacterial OTUs in all samples. The original data of bacterial OTUs quantity, involved seven samples (seven replicates for each sample) and a total of 49 original data.

10.7717/peerj.14359/supp-5 Supplemental Information 5 The statistics of fungal OTUs in all samples. The original data of fungal OTU quantity, involved seven samples (seven replicates for each sample) and a total of 49 original data.

10.7717/peerj.14359/supp-6 Supplemental Information 6 Relative abundance of bacteria in all samples. The relative abundance of major bacteria in soil microorganisms, involved seven samples (seven replicates for each sample) and a total of 49 original data.

10.7717/peerj.14359/supp-7 Supplemental Information 7 Relative abundance of fungi in all samples. The relative abundance of major fungi in soil microorganisms, involved seven samples (seven replicates for each sample) and a total of 49 original data.

10.7717/peerj.14359/supp-8 Supplemental Information 8 The soil physical and chemical properties of all samples. The soil physical and chemical properties, involved seven samples (seven replicates for each sample) and a total of 49 original data.

## Additional Information and Declarations

### DNA Deposition

The following information was supplied regarding the deposition of DNA sequences: The Illumina sequencing data is available at NCBI: PRJNA857463 (fungi), and PRJNA857576 (bacteria).

### Data Availability

The following information was supplied regarding data availability: The raw measurements are available in the Supplemental Files.
