# Supplementary figures and images for: Soil properties under different ecological restoration modes for the quarry in Yanshan mountains of Hebei province, China
Source: PeerJ. 2022 Nov 18;10:e14359. doi: 10.7717/peerj.14359 (PMC9677879; doi:10.7717/peerj.14359)

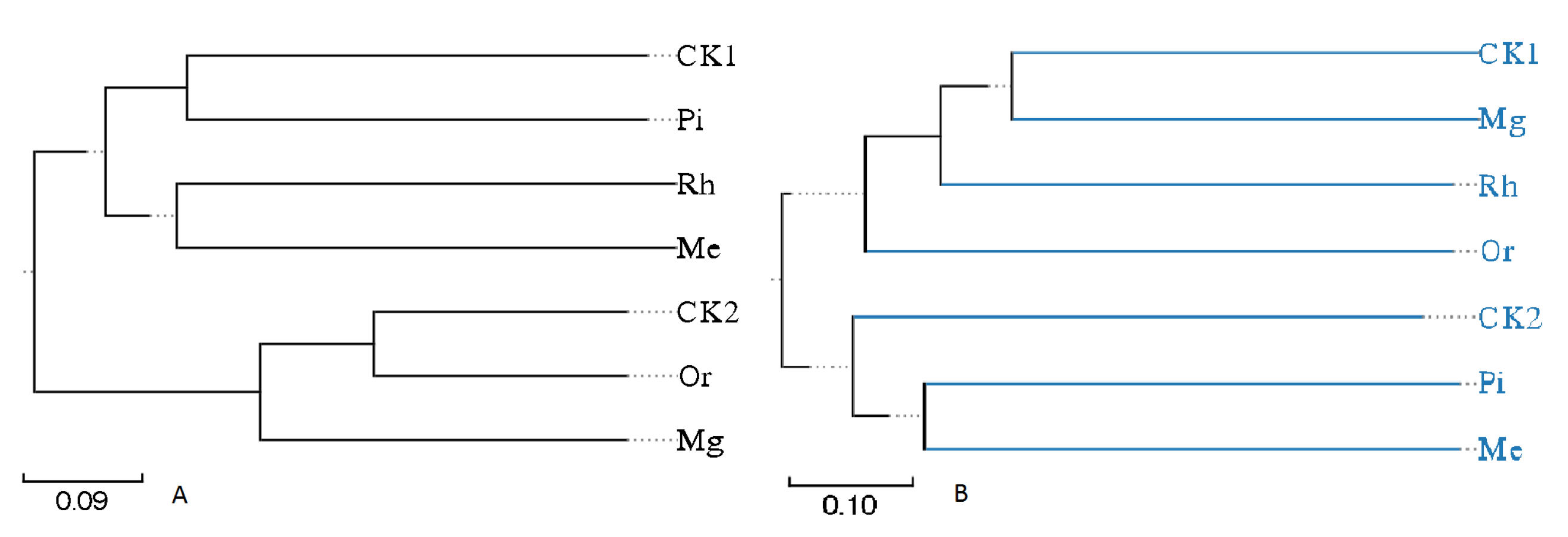

Supplement: Supplemental Information 1 [file peerj-10-14359-s001.png]

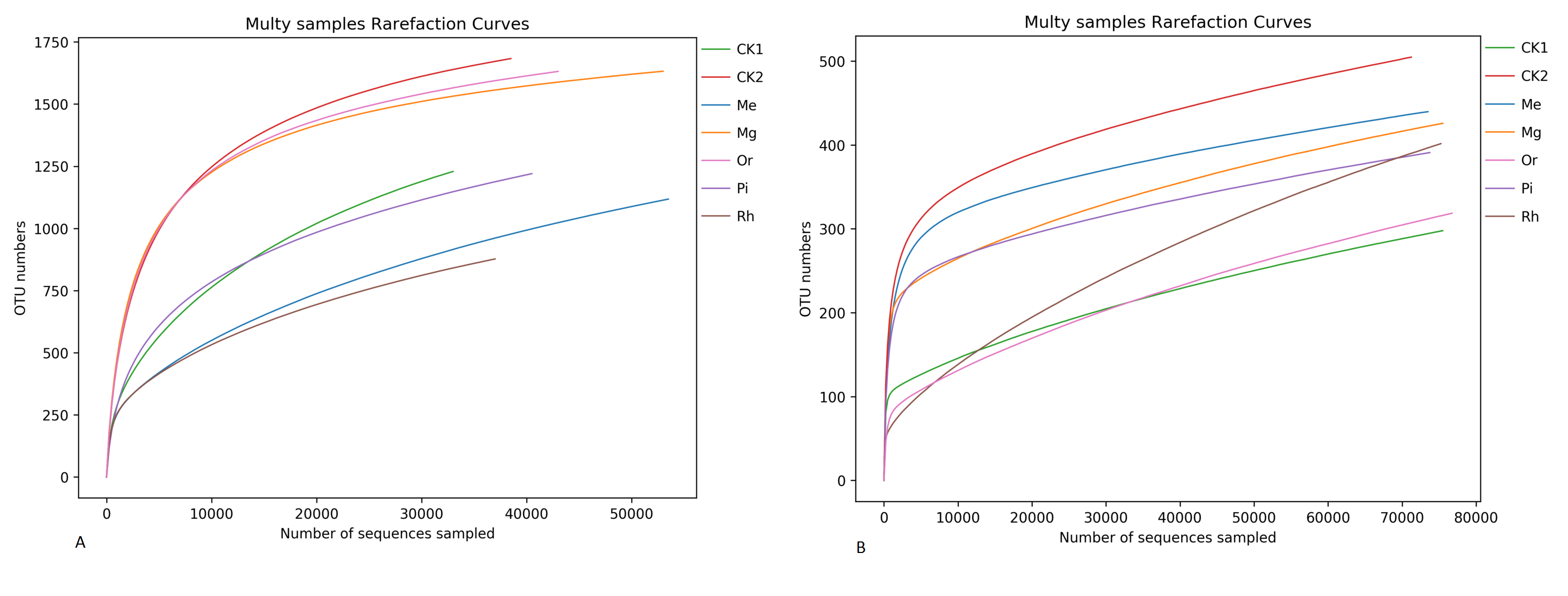

Supplement: Supplemental Information 2 — Rarefaction curves about the OTUs reached the plateau with the increase of the number of sequences per sample, stressing that the 35,000 and 20,000 sequences for each sample were sufficient to characterize soil bacterial and fungal communities in the studied soils. [file peerj-10-14359-s002.png]

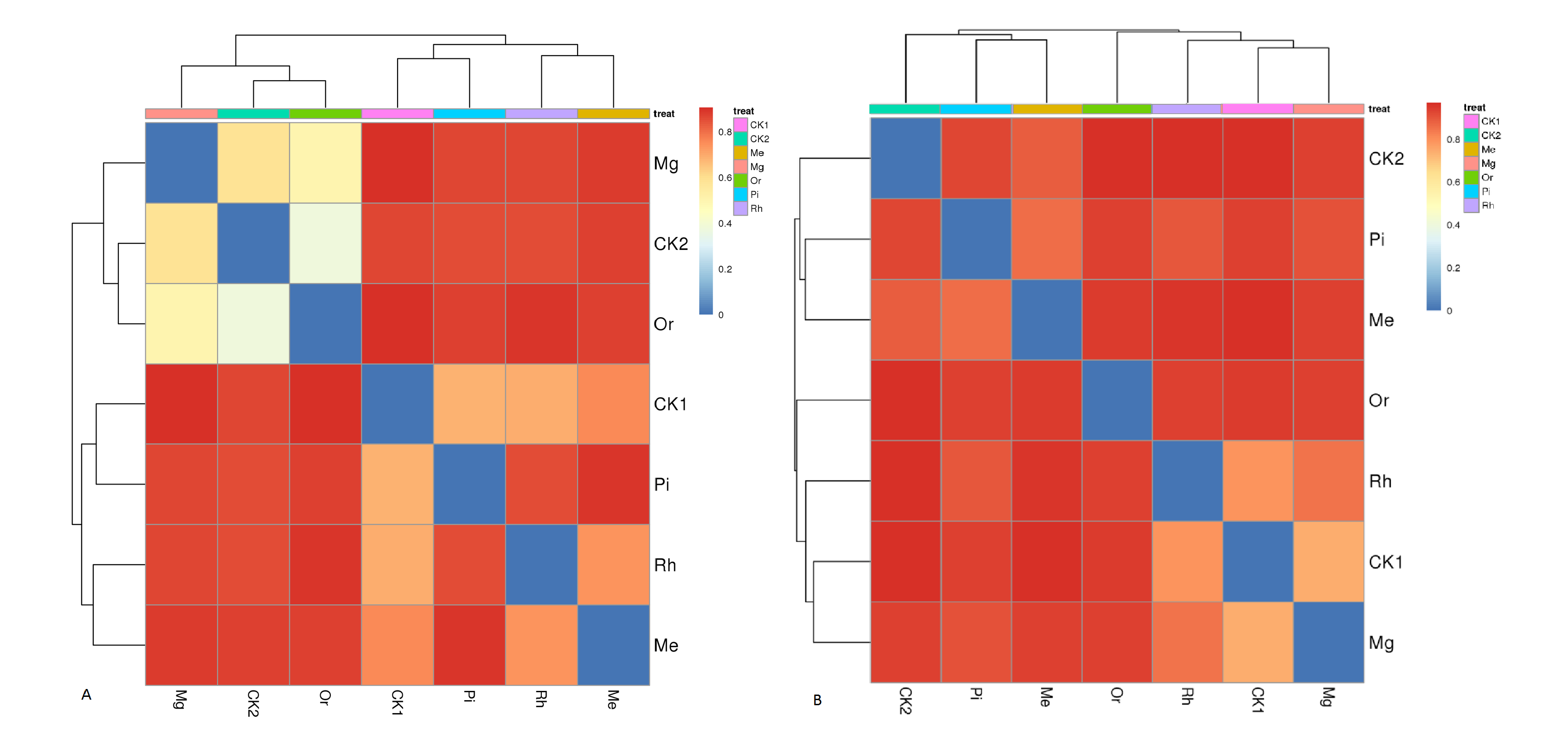

Supplement: Supplemental Information 3 — The heatmap based on bray distance indicated that the soil bacterial community structure of seven plots could be divided into five relatively concentrated clusters. [file peerj-10-14359-s003.png]
